# Vaccination Coverage and Prevention Counselling for Vaccine‐Preventable STIs Among HIV PrEP Users in São Paulo, Brazil: A Retrospective Cohort Study

**DOI:** 10.1002/jia2.70152

**Published:** 2026-07-24

**Authors:** Marjorie Marini Rapozo, Amanda Nazareth Lara, Victor Cabelho Passarelli, Laísa Rivas Dapousa Ramos, Patricia Silva Montes, Camila de Melo Picone, Angela Carvalho Freitas

**Affiliations:** ^1^ Serviço de Extensão ao Atendimento de Pacientes HIV/AIDS (SEAP), Divisão de Doenças Infecciosas e Parasitárias Hospital das Clinicas HCFMUSP, Faculdade de Medicina, Universidade de Sao Paulo Sao Paulo Brazil

**Keywords:** hepatitis A, hepatitis B, human papillomavirus, pre‐exposure prophylaxis, vaccines

## Abstract

**Introduction:**

HIV pre‐exposure prophylaxis (PrEP) users may be disproportionately vulnerable to sexually transmitted infections (STIs) in general, including several that are vaccine‐preventable. Understanding immunization patterns in this population is, therefore, crucial. However, data on vaccination coverage among Brazilian PrEP users remains limited.

**Methods:**

We conducted a retrospective single‐centre study of adults using HIV PrEP at an STI clinic in São Paulo, Brazil, between 2017 and 2024, to assess vaccination adequacy for vaccine‐preventable STIs among PrEP users, as well as other STI prevention measures during follow‐up. Demographic characteristics, substance use, STI history and vaccination status for hepatitis A (HAV), hepatitis B (HBV), human papillomavirus (HPV) and MPox were extracted from medical records, immunization registries and laboratory results, and descriptive analyses were performed.

**Results:**

Among 190 participants (median age: 36 years), 89.5% were gay or other men who have sex with men (MSM). Over a mean follow‐up period of 45 months, complete vaccination coverage was observed in 97.7% for HBV, 49.5% for HAV, 24.2% for HPV and 1.6% for MPox. Despite documented prior vaccination, a proportion of participants remained susceptible to HAV (16.3%) and HBV (2.3%). Furthermore, a substantial proportion of participants (32.1% for HAV, 53.7% for HPV and 81.0% for MPox) had neither a documented vaccination status nor a provider recommendation for vaccination recorded in their medical charts. HPV‐ and MPox‐related clinical lesions were documented in 17.9% and 2.1% of participants, respectively.

**Conclusions:**

Notable gaps in immunization against preventable STIs were observed in this PrEP cohort in São Paulo, Brazil. While HBV coverage was high, uptake of HAV, HPV and MPox vaccines was low. Addressing these gaps in our cohort requires transitioning from mere provider recommendations to structural public health policies. Implementing on‐site vaccine administration within PrEP services, integrating immunization into STI screening, expanding free access and addressing key vulnerabilities are critical steps to eliminate structural barriers and reduce the STI burden.

## 1 Introduction

Pre‐exposure prophylaxis (PrEP) is an effective pharmacological strategy that reduces the risk of HIV acquisition, particularly among the most vulnerable populations [1, 2]. In Brazil, PrEP is distributed by the Brazilian public health system (“Sistema Único de Saúde”; “SUS”) as a fixed‐dose combination tablet containing 300 mg of tenofovir disoproxil fumarate and 200 mg of emtricitabine, which can be used either as a daily oral regimen or on demand, which is suitable for men who have sex with men (MSM) and for transgender women who are not using gender‐affirming estrogen [3].

The global expansion of PrEP use has been associated with an increased incidence of sexually transmitted infections (STIs) [4], including those preventable by immunization, such as hepatitis A (HAV), hepatitis B (HBV) and human papillomavirus (HPV). However, the impact of such infections and related preventive measures remains insufficiently addressed in the context of PrEP use, particularly in low‐ and middle‐income countries.

Several cohort studies from high‐income countries estimate that among PrEP users, susceptibility to HAV may exceed 50%, whereas susceptibility to HBV, due to widespread vaccination, is typically below 30%. Regarding HPV vaccine coverage, up to 98% of PrEP users have not been previously vaccinated, and the proportion of immunized individuals increases among younger age groups [5, 6, 7, 8].

In Brazil, HBV vaccination is universal. It is scheduled in four doses (at birth, 2, 4 and 6 months of age), while older, unvaccinated individuals require a three‐dose primary series. HAV vaccination, introduced in 2014 in the public health system, consists of a single dose at 15 months of age. However, adolescents and young adults remain largely unprotected [9]. HAV vaccination was offered free of charge as part of a campaign targeted at gay and other MSM in São Paulo between May 2018 and June 2023, following a concentrated outbreak in this population [10]. More recently, after a 2‐year hiatus and emerging evidence of a new outbreak among MSM in several major Brazilian cities [11], the programme was reinstated and expanded nationwide.

In addition, national uptake of the quadrivalent HPV vaccine remains limited (81.2% for girls aged 9−14 years and 69.0% for boys aged 9−14 years) [12], despite its availability through the National Immunisation Program (PNI) for adolescents since 2014. As of 3 July 2024, PrEP users aged 15−45 years have also been included in the targeted population for the three‐dose schedule through the PNI [13]. While the PNI distributes exclusively the quadrivalent HPV vaccine, the nonavalent HPV vaccine is available only at private clinics.

Following the 2022 MPox outbreak, the Jynneos vaccine received regulatory approval and became available in Brazil on a limited basis. Despite PrEP users being eligible through the national programme, recurrent shortages [14] left many unvaccinated, as priority was given to people living with HIV and CD4 counts ≤ 200 cells/mm^3^.

Vaccine‐preventable STIs remain a public health concern among MSM in general, particularly among those using PrEP. Given the limited availability of data and the absence of national studies on vaccine coverage in this population, we aimed to assess the vaccination adequacy of PrEP users regarding vaccine‐preventable STIs during follow‐up at a PrEP clinic. Additionally, we evaluated the adequacy of preventive care for STI‐related conditions in this population (combined prevention strategies, harm reduction practices and vaccine‐related counselling).

## 2 Methods

This is a descriptive study with a retrospective design. It was carried out at SEAP, an HIV, STI and PrEP clinic in São Paulo, Brazil, part of the largest hospital complex in Latin America. Medical records, the Hospital Immunisation Registration System (SI‐CRIE) and the National Immunisation Program Vaccine Registry (SI‐PNI) were used as data sources.

The local Research Ethics Committee approved the study (number 7.022.774). All participants under active follow‐up provided written informed consent prior to study participation. For participants lost to follow‐up, the ethics committee granted a waiver of written informed consent.

Inclusion criteria consisted of individuals aged 18 years or older who received PrEP care at our clinic, encompassing patients under active follow‐up and those who were subsequently lost to follow‐up. Participants were excluded if they failed to collect their medication from the institutional pharmacy, could not be contacted during the inclusion period (if they were under active follow‐up) or had been using PrEP for less than 6 months. Individuals were considered lost to follow‐up if they discontinued care for at least 6 months between January 2023 and July 2024, after completing the minimum required 6‐month initial follow‐up period.

The sample size was calculated based on the total population of 369 active PrEP users at the service during the study period (2023–2024). To estimate prevalence with a 95% confidence interval, we assumed an anticipated prevalence of 50% for the primary outcomes—a conservative approach that maximizes the required sample size—and a 5% margin of error. This resulted in a required minimum sample size of 189 participants. Descriptive analyses included medians, interquartile ranges (IQR) and proportions. All statistical analyses were performed using Stata 13.1 (StataCorp, College Station, TX).

Data was collected using a structured form via the REDCap platform [15], with direct data entry into the electronic database. Sources included the Electronic Patient Record (EPR), SI‐CRIE and SI‐PNI records. Data collected exclusively from EPR included sociodemographic characteristics, use of psychoactive substances (PAS), chemsex (defined as the intentional use of specific PAS immediately before or during planned sexual activity to facilitate, enhance or prolong the experience) [16, 17] and prior history of STIs. Vaccination status for HAV, HBV, HPV and MPox was cross‐referenced across three databases: EPR, SI‐CRIE and SI‐PNI. We acknowledge that EPR data quality depends on patients presenting vaccination certificates for manual entry by clinicians, while national systems (SI‐CRIE and SI‐PNI) may have registry lags. To ensure data completeness and avoid underestimating coverage, we prioritized the source providing the most comprehensive documentation; specifically, the most complete vaccination schedule found across all sources was recorded.

Full vaccination was defined as the recorded completion of two doses for HAV and MPox, and three doses for HBV and HPV. Recommendation to vaccinate against such conditions was also evaluated.

HAV susceptibility was defined as the absence of anti‐HAV antibodies (both IgM and IgG), and prior contact or vaccination was defined as the presence of detectable anti‐HAV IgG with negative IgM. HBV susceptibility was defined as the absence of both anti‐HBc and anti‐HBs antibodies; vaccine‐induced immunity was defined by negative anti‐HBc and anti‐HBs ≥10 IU/mL, prior acquisition was defined by anti‐HBc positivity with or without anti‐HBs ≥10 IU/mL, and active HBV infection was defined by the presence of HBsAg and/or HBeAg. Chronic HBV infection was defined as the presence of detectable HBsAg for more than 6 months. Hepatitis C (HCV) diagnosis was defined as either seroconversion during the study period or detectable HCV‐RNA in individuals with a prior history of HCV acquisition. MPox diagnosis was determined by the annotation of such a condition in the medical records.

## 3 Results

### 3.1 Demographic Characteristics and Vulnerability Profile for STIs

**FIGURE 1:**
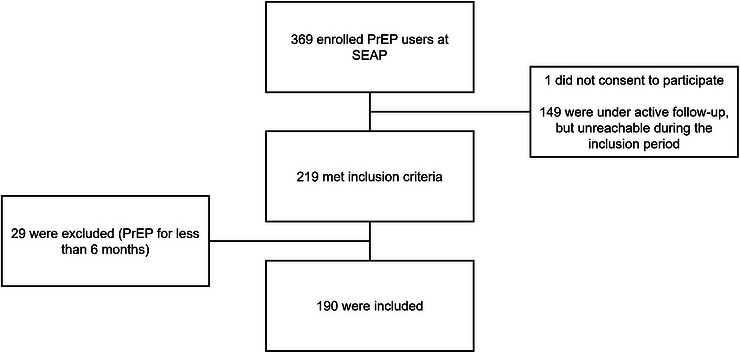
Sample inclusion criteria flow chart. Abbreviation: PrEP, pre‐exposure prophylaxis.

One hundred and ninety participants met the inclusion criteria (Figure 1). The median follow‐up time was 45.4 months (interquartile range: 18.4−65.1 months). The median age of the participants was 36 years (interquartile range: 32−42 years). Most participants were gay men and other MSM (170; 89.5%), of white ethnicity (149; 78.4%) and had 12 or more years of formal education (166; 87.4%). Black and mixed‐race individuals represented 11.1% (21) of the study population (Table 1).

**TABLE 1:** Sociodemographic profile and use of alcohol and/or other psychoactive substances (PAS) among PrEP users at SEAP.

Variable	Median (IQR)/*n* (%)	Variable	*n* (%)
**Age (years)**	37.7 (32; 42)	**Race**	
**Follow‐up (months)**	45.4 (18.4; 65.1)	White	149 (78.4)
**Assigned sex at birth**		Black	7 (3.7)
Male	183 (96.3)	Brown	14 (7.4)
Female	7 (3.7)	Asian	16 (8.4)
**Gender identity**		Indigenous	2 (1.0)
Cisgender	183 (96.3)	**Transactional sex practices**	
Transgender	1 (0.5)	Yes	7 (3.7)
Non‐binary	1 (0.5)	No	2 (1.0)
Other	1 (0.5)	No answer	181 (95.3)
No answer	4 (2.1)	**Psychoactive substance use** ^a^	123/190
**Education**		Alcohol	84 (68.3)
4−7 years	4 (2.1)	Cocaine	24 (19.5)
8−11 years	13 (6.8)	Poppers	6 (4.9)
12 or more years	166 (87.4)	Methanphetamine	1 (0.8)
No answer	7 (3.7)	Ketamine	2 (1.6)
**Sexual orientation**		GHB	5 (4.1)
Homosexual	158 (83.2)	Cannabis	50 (40.7)
Bisexual	12 (6.3)	Crack	1 (0.8)
Heterosexual	14 (7.4)	Ecstasy	31 (25.2)
Other	2 (1.0)	LSD	7 (5.7)
No answer	4 (2.1)	Other	23 (18.7)

Abbreviations: GHB, gamma‐hydroxybutyrate; LSD, lysergic acid diethylamide.

^a^Specific substance percentages are calculated based on the total number of users (*n* = 123).

Overall, alcohol and/or other PAS use was recorded for 123 of the 190 participants (64.7%). The most frequently reported substance was alcohol in any amount, documented for 84/123 participants (68.3%), followed by cannabis (50/123; 40.7%), ecstasy (31/123; 25.2%) and cocaine (24/123; 19.5%). When assessed independently, chemsex use was documented for 15 participants (7.9%), with cocaine being the most frequently used substance in this subgroup (seven reports), followed by poppers/volatile nitrites (six reports) and gamma‐hydroxybutyrate (GHB; five reports) (Table 1).

### 3.2 Occurrence of Viral STIs

Regarding viral hepatitis, two asymptomatic cases of hepatitis A were identified, diagnosed through seroconversion unrelated to vaccine administration. No new cases of hepatitis B were documented. Moreover, two cases of HCV acquisition (1.1%) were recorded during follow‐up.

As for HPV clinical occurrences, 28 cases (14.7%) were documented during follow‐up. In 21 records (11.1%), the absence of HPV‐related lesions was explicitly noted, and in 131 of the 190 medical records evaluated (69.0%), there was no mention of HPV lesions.

Regarding the incidence of MPox, four cases (2.1%) were recorded among the 190 participants.

### 3.3 Vaccination Coverage and Prevention Counselling for Vaccine‐Preventable STIs

#### 3.3.1 Hepatitis A

Complete vaccination against HAV was recorded for 94 of the 190 participants (49.5%), 51 (26.8%) at baseline and 43 (22.6%) during follow‐up. Six participants (3.2%) had incomplete vaccination, and 29 (15.3%) had not received any HAV vaccine. Vaccination records were absent for 61 participants (32.1%). None of the six participants with incomplete HAV vaccination seroconverted. No serological data were available for eight participants (4.2%).

Among the 28 susceptible participants, 16 (57.1%) did not receive the vaccine despite documented provider recommendations, while 12 (42.9%) had no recorded recommendation in their files.

#### 3.3.2 Hepatitis B

Regarding HBV, vaccine uptake was analysed among the 177 participants for whom vaccination was clinically indicated (excluding 11 with prior natural immunity and two with chronic HBV). Among these eligible participants, 173 (97.7%) had completed the full vaccination schedule by the end of follow‐up, with 23 completing it during the study period. Four participants (2.3%) remained partially vaccinated and susceptible. Overall, 151 (85.3% of the eligible cohort) demonstrated vaccine‐induced immunity. Among the nine participants who required new or booster doses during follow‐up, seven (77.8%) received documented counselling but did not subsequently complete the vaccination schedule.

#### 3.3.3 HPV

Complete HPV vaccination was recorded for 46 of the 190 participants (24.2%). An additional 42 participants (22.1%) had incomplete vaccination (one or two doses); for 40 of these (95.2%), the most recent dose was administered during follow‐up, while only one participant had clearly initiated the series prior to entry. No HPV vaccination records were available for 102 participants (53.7%).

Among the 143 participants who had not completed vaccination until the end of the study and had no history of HPV‐related lesions, vaccination was recommended for 48 of them (33.6%).

#### 3.3.4 MPox

There were only three (out of 190) records (1.6%) of complete MPox vaccination (including one participant who reported receiving two doses abroad) and one record of incomplete immunization. All recorded MPox vaccinations occurred after the initiation of PrEP follow‐up. Of 154 records (81.0%), no specific data on MPox vaccination were available; 33 records (17.4%) reported receiving no MPox vaccine.

Vaccination recommendations were recorded for three participants, none of whom were subsequently vaccinated or acquired MPox. Conversely, one vaccinated participant had no prior recommendation recorded in the EPR.

**FIGURE 2:**
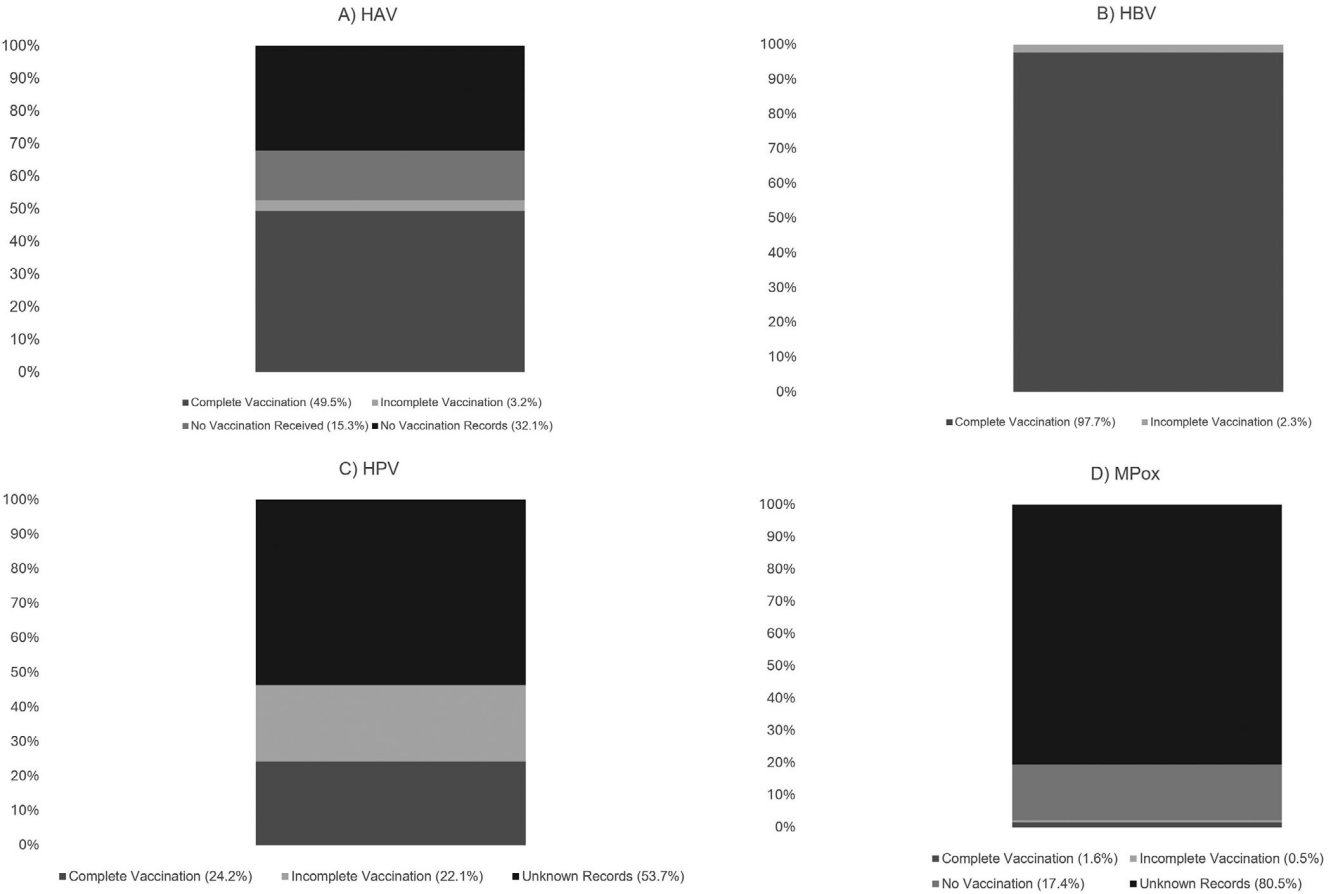
Vaccination status among PrEP users at SEAP. Vaccination status for hepatitis A (HAV) (A), hepatitis B (HBV) (B), human papilloma virus (HPV) (C) and MPox (D). Vaccination status for hepatitis B was focused on vaccine‐eligible individuals.

Figure 2 graphically represents the STIs vaccination status among PrEP users.

## 4 Discussion

It is well established in the literature that PrEP use can be associated with increased incidence of STIs in general [4, 18, 19, 20], likely secondary to an increased sense of security regarding the low risk of HIV acquisition. Moreover, although recent studies have shown a trend towards stabilization or even a decline in STI incidence after 1 year of PrEP use [21, 22], vaccine‐preventable STIs continue to represent a significant and potentially underaddressed burden in this population, particularly given the suboptimal vaccination coverage observed among PrEP users [6, 23], which may reflect an important gap in the PrEP care continuum.

Our study identified a high anti‐HAV seroprevalence (>80%), likely reflecting both past exposure among older participants and the targeted provision of hepatitis A vaccination to MSM by the Brazilian public health system in São Paulo between May 2018 and June 2023, following a concentrated outbreak in this population. Notably, while all individuals who completed vaccination during follow‐up achieved seroconversion, none of the partially vaccinated participants did so, underscoring the importance of the full two‐dose schedule for effective protection against HAV acquisition and severe disease.

International comparisons highlight substantial variability in susceptibility across PrEP populations. In France—where hepatitis A vaccination is not universally offered—susceptibility ranged from 42.3% to 50.4% in two cohorts of PrEP users [5, 6]. Conversely, Italy, which adopted universal HAV vaccination, reported a lower susceptibility rate of 20.3%, although only 21.5% of participants had previously received immunization [7]. In Brazil, a cross‐sectional study in the northeastern state of Rio Grande do Norte reported an HAV susceptibility of 37.6% among PrEP users [24]. A Dutch study in 2019 similarly reported a 37% seroprevalence among MSM and noted higher uptake when vaccination was provided free of charge [25].

Importantly, Brazil has recently expanded its policy: since 29 April 2025, HAV vaccination has been offered nationwide to all PrEP users in response to a new outbreak among MSM [11]. This broader eligibility may substantially improve coverage and reduce susceptibility over time.

Regarding hepatitis B, a retrospective Italian study found a 0.46% prevalence of HBsAg positivity among PrEP users [26], whereas the present study reported a rate of 1.1%. This relatively low prevalence may be attributed to Brazil's universal hepatitis B vaccination programme, which began in 2016, as well as to the inclusion of infants under 1 year of age as a target population since 1997, thereby contributing to the high vaccine coverage observed in this study. Nevertheless, socioeconomic factors still influence vaccine uptake, with a greater susceptibility to HBV acquisition among PrEP users, especially those with socioeconomic vulnerabilities [27].

Despite being highly prevalent among MSM using PrEP [28], our study revealed relatively few records of HPV‐related lesions, likely due to the need for physical and genital examination, which is not always conducted during medical consultations. An Italian study [29] showed that anal HPV detection, especially with high‐risk genotypes, was associated with recreational drug use and the presence of at least two concurrent STIs. Such characteristics are similar to the pattern observed in our cohort, and reinforce the importance of complete coverage of the HPV vaccine in this population. In contrast to this argument, we observed relatively low adherence to the three‐dose regimen against HPV, likely due to the short time between the vaccine's inclusion in Brazil's national immunization programme for PrEP users aged 15−45 years and the data acquisition period. Moreover, despite availability in the private sector for several years, its high cost still limits access to many, as also shown in countries such as Canada and the United States [30, 31]. Still, with the expansion of HPV vaccine eligibility criteria through the Brazilian public system as of July 2024 [11], coverage is expected to improve shortly.

In 2022, the global MPox epidemic disproportionately affected MSM [32], some of whom were among the participants from our study. Additionally, while natural immunity from prior MPox infection was observed in some participants, we could not precisely quantify these cases, as they often occurred between scheduled visits and were inconsistently recorded in medical records. This likely led to an underestimation of the total number of participants with some degree of immunity. Unfortunately, the amount of Jynneos vaccine doses acquired by Brazil's Ministry of Health was insufficient to cover this vulnerable population due to limited distribution and a short availability period. This resulted in extremely low vaccine coverage among PrEP users, as well as in a low rate of vaccination recommendation by healthcare professionals, showing there is a large space for improvement.

Furthermore, the high prevalence of substance use—especially chemsex‐related—further highlights the importance of addressing such behaviours directly during prevention consultations to guide harm‐reduction strategies [33]. Yet, only 8% of medical records documented substance use explicitly. Besides, merely 4% mentioned transactional sex or sex work. These omissions suggest that key vulnerabilities are not being consistently identified or discussed. Together, these findings point to clear opportunities for improvement at both the local and national levels.

Our study has several strengths—one of the main ones being that it is one of the few Brazilian investigations to evaluate both vaccination status and clinician‐led vaccination recommendations among PrEP users. This dual perspective not only reveals existing gaps but also helps determine whether current practices are meeting expected standards. Our findings point to several challenges. The limited availability of some vaccines at the governmental level—exemplified by the MPox vaccine shortage—may contribute to the risk of new outbreaks. Moreover, the persistently low vaccination coverage, even when healthcare professionals recommended vaccines, suggests the need for targeted public health campaigns, particularly for hepatitis A and HPV. The substantial number of participants with current or past HBV and acute HCV infection further highlights the vulnerability of MSM to viral hepatitis [34]. Additionally, the lack of seroconversion for hepatitis B observed in some individuals indicates that continuous monitoring for HBV acquisition may be necessary, even if considering that daily and event‐driven PrEP may offer protection against HBV in MSM as well [35].

Our findings must be interpreted in consideration of the limitations of our study. We analysed data from a single centre in São Paulo, and the retrospective nature of the study, with varying follow‐up durations, may have led to missing data for some variables due to its reliance on existing medical records. Furthermore, the exclusion of participants with less than 6 months of follow‐up may have limited our ability to assess the immediacy of vaccine initiation and the care provided to those who did not remain in long‐term PrEP follow‐up. Also, the study period (2017−2024) encompasses the COVID‐19 pandemic, which likely influenced sexual behaviour and healthcare utilization. Our retrospective review found inconsistent documentation of this impact: while some patients reported reduced social interaction due to fear of infection, others showed no recorded changes in behaviour. Due to the lack of standardized data on this variable, the pandemic's specific influence on our observed trends remains a contextual limitation. Moreover, our study population consisted predominantly of highly educated, high‐income MSM, which may limit the generalizability of our findings. In particular, the small number of participants from other sexual and gender minority groups, including female sex workers and transgender individuals, highlights the persistent barriers such populations face in accessing healthcare.

Additionally, a major operational limitation of our service is the lack of on‐site vaccine administration for any of the STIs evaluated due to structural constraints. Because patients must be referred to external public or private immunization clinics, this required displacement acts as a significant barrier to care. Consequently, the lower uptake observed for certain vaccines likely reflects these navigation obstacles within the healthcare system rather than a simple lack of patient adherence.

Importantly, our data show high adherence to vaccine recommendations, especially when offered universally and free of charge. Therefore, facilitating access, whether by expanding vaccine availability through the public health system or reducing costs in the private healthcare setting, could increase vaccine coverage and may be particularly promising for improving the alignment between healthcare providers’ recommendations and patients’ willingness to receive vaccines [36].

## 5 Conclusions

Gaps in vaccine coverage and the presence of vulnerabilities, particularly PAS use, reinforce the need for integrated strategies that combine government‐supported public health policies, healthcare provider training and patient awareness campaigns regarding the importance of STI prevention with vaccines, and the implementation of on‐site vaccine administration directly within PrEP services may represent a crucial step to overcome logistical barriers and eliminate missed clinical opportunities. Going beyond traditional HIV prevention methods by establishing a single‐point‐of‐care model is essential to expand equitable access to comprehensive sexual healthcare and improve uptake of available preventive interventions.

## Author Contributions

MMR collected, revised, and analysed the data and wrote the manuscript. ANL analysed the data and revised the manuscript. VCP wrote and revised the manuscript. LRDR and PSM revised the manuscript. CMP collected and organized data. ACF designed the research study, analysed the data, wrote and revised the manuscript. All authors have read and approved the final version of the manuscript for publication.

## Conflicts of Interest

The authors declare that they have no competing interests or other interests that might be perceived to influence the results and/or discussion reported in this paper.

## Data Availability

The data that support the findings of this study are available from the corresponding author upon reasonable request.
